# Elements in the 5′ Untranslated Region of Viral RNA Important for HIV Gag Recognition and Cross-Packaging

**DOI:** 10.3390/v17040551

**Published:** 2025-04-10

**Authors:** Zetao Cheng, Olga A. Nikolaitchik, Alice Duchon, Jonathan M. O. Rawson, Vinay K. Pathak, Wei-Shau Hu

**Affiliations:** 1 Viral Recombination Section, HIV Dynamics and Replication Program, National Cancer Institute, Frederick, MD 21702, USA; zetao.cheng@nih.gov (Z.C.); nikolaio@mail.nih.gov (O.A.N.); alice.duchon@nih.gov (A.D.); jonathan.rawson@fda.hhs.gov (J.M.O.R.); 2 Viral Mutation Section, HIV Dynamics and Replication Program, National Cancer Institute, Frederick, MD 21702, USA; pathakv@mail.nih.gov

**Keywords:** HIV-1, HIV-2, Gag, RNA packaging, 5′ untranslated region, Gag binding site, unpaired guanosine

## Abstract

During retrovirus assembly, Gag packages unspliced viral RNA as the virion genome. Genome packaging is usually specific with occasional exceptions of cross-packaging RNA from distantly related retroviruses. For example, HIV-1 Gag can efficiently package HIV-2 RNA. To better understand how HIV-1 Gag selects packaging substrates, we defined elements in the HIV-2 5′ untranslated region (UTR) that are important for this process. Although sharing little homology, both HIV-1 and HIV-2 5′ UTRs have unpaired guanosines essential for packaging by their own Gag. Simultaneously substituting guanosines of nine sites in the HIV-2 5′ UTR caused severe defects in HIV-1 Gag-mediated packaging. Two of the nine sites are particularly important, mutating each one impaired HIV-1 Gag-mediated packaging, whereas the other sites required mutations in multiple sites to produce similar effects. Additionally, we identified one site that impacts HIV-1 Gag but is dispensable for HIV-2 Gag selective packaging. Furthermore, combining mutations has an additive effect on packaging defects for HIV-1 Gag, in contrast to the previously reported synergistic effects for HIV-2 Gag. Our study demonstrates that Gag proteins from two different retroviruses recognize and use mostly the same set of cis-acting elements to mediate RNA packaging and provide the mechanistic basis for genome cross-packaging.

## 1. Introduction

Retroviruses package two copies of full-length viral RNA into virions to carry the genetic information essential for continuing replication to the next host [1,2]. Thus, RNA genome packaging is a critical step in generating infectious viruses. Full-length HIV-1 RNA is only a minor RNA species in infected cells, and yet most HIV-1 virions contain viral unspliced RNA, indicating that genome packaging is a highly selective and efficient process [3]. Mutation analyses have shown that domains in the Gag polyprotein and elements in full-length viral RNA, especially those in 5′ untranslated regions (UTRs), mediate RNA genome packaging [4,5,6,7,8,9]. However, many aspects of viral genome packaging are not understood, including how Gag selects viral RNA to achieve highly efficient genome packaging.

HIV-1 and HIV-2 are human pathogens that can each cause AIDS. Although both are zoonotic viruses, HIV-1 and HIV-2 originated from different nonhuman primate lentiviruses [10]. HIV-1 was introduced into the human population through cross-species transmissions of simian immunodeficiency virus (SIV) from chimpanzees (SIVcpz) and gorillas (SIVgor) [11,12,13]. In contrast, HIV-2 originated from zoonotic infections by SIVs that infect sooty mangabeys (SIVsm) [14,15]. Genetically, HIV-1 (NL4-3) and HIV-2 (ROD12) share ~52% nucleotide sequence identity in their viral genomes and ~54% amino acid sequence identity in Gag. Although only distantly related, HIV-1 Gag can efficiently package HIV-2 RNA [16,17]. Intriguingly, this interaction is nonreciprocal, as HIV-2 Gag cannot package HIV-1 RNA [16,17].

The 5′ UTRs of HIV-1 and HIV-2 share little nucleotide identity, ~43% between molecular clones NL4-3 and ROD12. However, both 5′ UTRs fold into complex RNA structures [18,19,20,21,22,23,24]. Additionally, both 5′ UTRs contain exposed guanosines that are essential for RNA packaging [25,26]. Using chemical probing and a “reverse-footprinting” assay, several exposed guanosines in the HIV-1 5′ UTR have been identified as the major nucleocapsid (NC) protein binding sites in virions [27]. Additionally, purified NC proteins and Gag polyprotein have also been shown to bind the exposed guanosines of in vitro transcribed HIV-1 5′ UTR RNA [28,29,30]. The impact of these exposed guanosines on genome packaging has been examined using G-to-A substitution mutants and measuring their RNA packaging efficiencies in viruses generated from infected cells [25]. These studies showed that several exposed guanosines are critical for efficient RNA packaging; G-to-A substitutions of these guanosines cause a drastic reduction in HIV-1 RNA packaging. Compared to HIV-1, the NC binding sites of the HIV-2 5′ UTR have not been as well characterized using biochemical or biophysical methods. However, exposed guanosines were identified based on RNA structures and the impact of substitution mutations on RNA packaging was examined [22,26]. Indeed, several exposed guanosines in the HIV-2 5′ UTR play key roles in mediating RNA packaging by HIV-2 Gag [26]. Therefore, the exposed guanosines in both the HIV-1 and HIV-2 5′ UTRs are important for packaging by their autologous Gag proteins.

Currently, it is not known how HIV-1 Gag recognizes and efficiently packages HIV-2 unspliced RNA. In this report, we examined whether HIV-1 Gag recognizes HIV-2 RNA using the exposed guanosines in the 5′ UTR and, if so, whether the same guanosines mediate packaging by two distinct Gag polyproteins. These studies provide new insights into how HIV-1 Gag selects RNA to be packaged into virions and also shed light on how cross-packaging occurs among distantly related retroviruses.

## 2. Materials and Methods

### 2.1. Plasmid Construction

The previously described HIV-2 plasmid pHIV-2-17T was derived from molecular clone pROD12, contains all the cis-acting elements required for viral replication, and expresses Tat, Rev, and a mouse thy1.2 gene in nef [31,32]. Additionally, pHIV-2-17T contains premature stop codons in gag and a truncated env. Plasmid PIH has the same general structure as pHIV-2-17T except that it harbors a different set of marker genes in nef including a puromycin resistance gene, an internal ribosomal entry site (IRES) from the encephalomyocarditis virus, and a mouse heat stable antigen (hsa) gene (puro-IRES-hsa). To introduce 5′UTR mutations, the NgoMIV-to-AgeI fragment of the PIH plasmid was replaced with DNA fragments containing mutations excised from previously described plasmids by standard molecular cloning techniques. WT-17T was derived from pHIV-2-17T with the following modifications: first the dimer initiation signal was altered from GGTACC to GCGCGC to reduce dimerization between the WT-17T and PIH RNAs. Additionally, near the 3’ end of gag contains a 12 bp (GCTTCCATCGAG) insertion, generating a polymorphic site that allows the detection and distinction of WT-17T or PIH RNA by allele-specific PCR.

An HIV-1 Gag- or HIV-2 Gag-expression vector was derived from pHAGE-SIN, a self-inactivating lentiviral vector that encodes an intact HIV-1 5′ LTR, a human cytomegalovirus (CMV) promoter, an IRES followed by a human B7 marker gene, and a truncated 3’ LTR with a 400 bp deletion in U3. HIV-1 or HIV-2 Gag was introduced downstream of the CMV promoter by standard molecular cloning techniques. In the HIV-2 Gag expressing vector, 27-bp nucleotide (CAG/AGG/GAG/ACA/CCA/TAC/AGG/GAG/CCA) downstream of the aforementioned polymorphic site in p6 was synonymously modified to CAA/AGA/GAA/ACT/CCT/TAT/AGA/GAA/CCT, to prevent its amplification in the allele-specific RT-PCR assay.

The general structures of all constructs were confirmed by restriction enzyme mapping and DNA sequencing of partial genomes.

### 2.2. Cell Culture, Generation of Cell Lines, Lentivirus Infection, and Flow Cytometry

Human embryonic kidney 293T cells were grown in Dulbecco’s modified Eagle’s medium supplemented with 10% fetal bovine serum, penicillin (100 U/mL), and streptomycin (100 μg/mL), and incubated in a humidified 37 °C incubator with 5% CO_2_. All transfections were performed using TransIT-LT1 Transfection Reagent (Mirus Bio, Madison, WI, USA) according to the manufacturer’s instructions. To generate the infectious virus, 293T cells were transfected with an HIV-2 construct (WT-17T or PIH) or SIN-Gag, together with two helper plasmids, pCMV∆8.2 and pHCMV-G. Plasmid pCMV∆8.2 expresses HIV-1 Gag/Gag-Pol and all of the accessory proteins, whereas pHCMV-G expresses vesicular stomatitis virus G protein (VSV G) [33,34]. Viral supernatants were harvested two days post-transfection, clarified through a 0.45 μm pore size filter to remove cellular debris, then used immediately or stored at −80 °C prior to infection.

To generate cell lines expressing two HIV-2 proviruses, 293T cells were first infected with the WT-17T virus at an MOI of <0.1, and infected cells were enriched by repeated cell sorting until >98% of the cells expressed the Thy marker. These singly infected cells were then infected with an HIV-2 PIH-derivative virus at an MOI of <0.1. One day after infection, cells were placed in supplemented media containing puromycin (1 μg/mL), generating a population of dually infected cells with >97% of cells expressing Thy1.2 and HSA markers. Each cell line contained at least 50,000 independent infection events.

The proportions of infected cells were determined by flow cytometry by detecting the expression of cell surface markers Thy1.2, HSA, and B7. To perform flow cytometry, cells were collected, washed twice with Dulbecco’s phosphate-buffered saline (DPBS) supplemented with 2.5% FBS, and then stained with allophycocyanin-conjugated anti-Thy1.2 antibodies (eBioscience, San Diego, CA, USA) and/or phycoerythrin-conjugated anti-HSA (BioLegend, San Diego, CA, USA), or anti-CD80 (B7) (BioLegend or Invitrogen, Carlsbad, CA, USA) for 30 min at 4 °C, followed by two additional washes using the same buffer. Flow cytometry was performed using an LSR II system (BD Biosciences, Franklin Lakes, NJ, USA), and cell sorting was performed on a FACSAria II system (BD Biosciences). Data obtained from flow cytometry were analyzed using FlowJo software V10.10.0 (FlowJo, LLC, Ashland, Wilmington, DE, USA).

### 2.3. RNA Isolation and Quantification, and Statistics

To measure RNA packaging efficiency, cell lines expressing two different HIV-2 proviruses were infected with an HIV-1 or HIV-2 Gag-expressing lentiviral vector, SIN-1Gag or SIN-2 Gag, respectively, in a 6-well plate. One day after infection, cells were gently lifted, and 20% of cells were placed in a new 6-well plate while 80% of the cells remained in the same plate. Cells in the new plate were used to measure the proportion of cells expressing B7 markers encoded by SIN-1Gag or SIN-2Gag, 3 days postinfection by flow cytometry. Cells in the same plate were used for cytoplasmic and virion RNA isolation. To maintain consistent experimental conditions, we used samples in which 15–35% of the cells were transduced by the SIN vectors and expressed B7 markers for analyses. After clarification through a 0.45 μm pore size filter (Millex), virion RNA was extracted using a QIAamp Viral RNA Mini kit (Qiagen, Hilden, Germany). Cytoplasmic RNA was isolated using a PARIS kit (Invitrogen) according to the manufacturer’s instructions. RNA was used for quantitative allele-specific RT-PCR immediately or stored at −80 °C. Quantitative allele-specific RT-PCR was performed with an iTaq Universal SYBR Green One-Step Kit (Bio-Rad, Hercules, CA, USA). Allele-specific forward primers INS-F (5′-TACTGCACCTCGAGGCTTCC-3′) and 2880-1F (5′-TTACTGCACCTCGAGCAGAG-3′) were designed to anneal to the sequence at the polymorphic site in the gag gene of 17T and the corresponding region in the PIH vector, respectively. Primer 2921R (5′-GTGGCTCCCTGTATGGTGTC-3′) annealed to both templates and was used as a primer for reverse transcription as well as the reverse primer for quantitative PCR. Virion and cytoplasmic RNAs were serially diluted for quantitative allele-specific RT-PCR, and the data points within the linear range were used. Each quantitative RT-PCR run included controls to measure cross-reactivity. We found that under the conditions used, the designed primers specifically detect the correct template, and the cross-reactivity was <0.01%. The packaging efficiency of PIH-derived RNA was calculated as the proportion of PIH RNA in the virion divided by the proportion of PIH RNA in the cytoplasm: [virion PIH/(virion PIH plus virion WT-17T)]/[cytoplasmic PIH/(cytoplasmic PIH plus cytoplasmic WT-17T)]. At least three independent experiments were performed for each mutant to measure RNA packaging efficiency.

### 2.4. Statistical Analyses

Statistical analyses were performed in GraphPad Prism v9.2.0. RNA packaging efficiencies were compared using a *t*-test, unpaired one-way ANOVA, or two-way ANOVA with a Bonferroni correction for multiple comparisons. Comparisons with *p* values less than 0.05 were considered statistically significant.

## 3. Results

### 3.1. Experimental System to Delineate Cis-Acting Elements in the HIV-2 5′ UTR That Are Important for HIV-1 Gag-Mediated RNA Packaging

**Figure 1:**
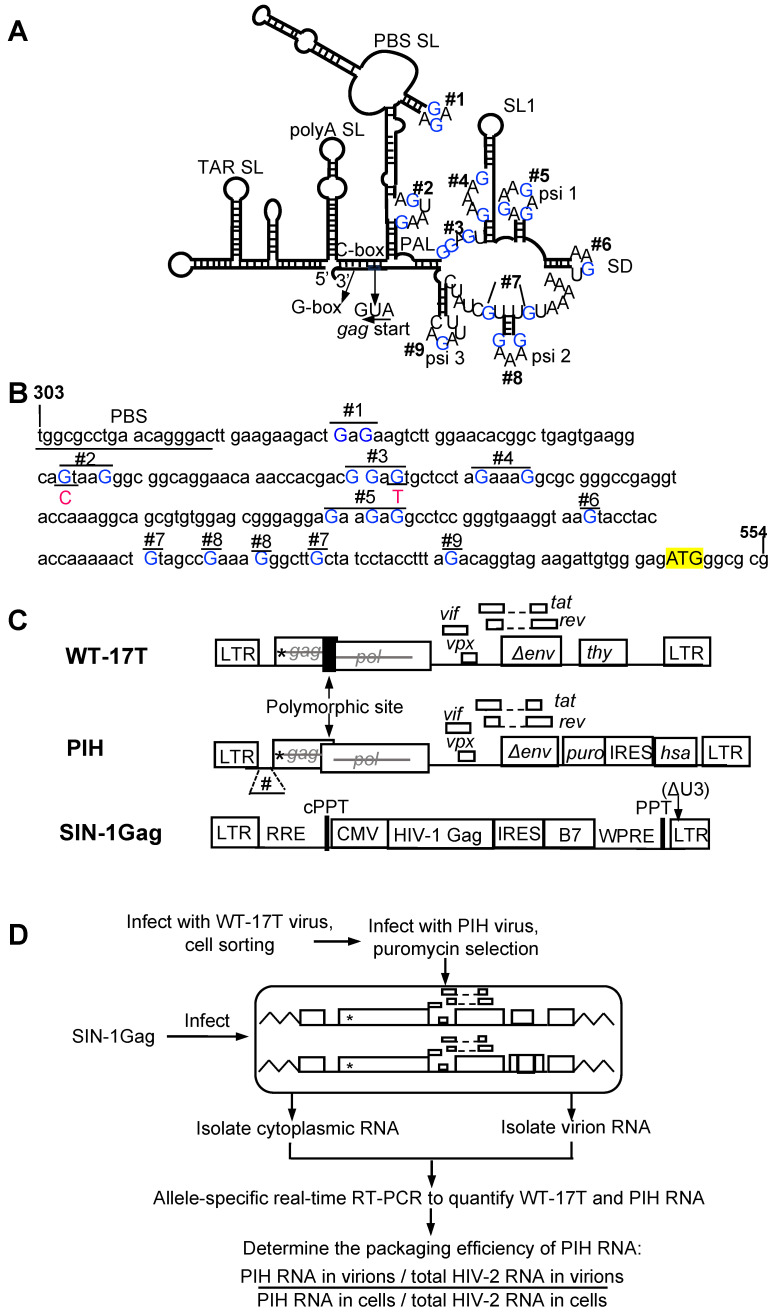
Experimental approach to study the role of unpaired guanosines in the HIV-2 5′ UTR on HIV-1 Gag packaging. (**A**) Schematic of the HIV-2 leader RNA structure as modeled by chemical probing studies [22] with unpaired guanosines indicated as blue “Gs”. (**B**) Mutations introduced in the HIV-2 5′ UTR. Mutated guanosines Gs are shown in blue uppercase. G-to-A substitutions were introduced in all mutations with two exceptions. One guanosine in site 2 and one guanosine in site 3 were mutated to C and T (indicated in red), respectively, to avoid the introduction of an unintended polyadenylation signal and translation start sites. The start codon “ATG” is highlighted in yellow. (**C**) General structures of HIV-2 constructs and the HIV-1 Gag-expressing lentiviral vector in this research. *, indicate inactivating mutations in *gag*; #, mutations in the 5′ leader. (**D**) Experimental protocol.

Among the multiple exposed guanosines in the HIV-2 5′ UTR (Figure 1A,B), some, but not all, play critical roles in the RNA genome packaging by HIV-2 Gag [26]. To determine whether HIV-1 and HIV-2 Gag polyproteins interact with the same sites in the HIV-2 5′ UTR, we established a system to examine the HIV-1 Gag packaging of HIV-2 RNA. In this system, each stable cell line harbors two HIV-2 proviruses, one containing all the guanosines in the 5′ UTR (WT-17T) and the other provirus (PIH) containing either an unmodified 5′ UTR (PIH-WT) or a mutated 5′ UTR. We then introduced HIV-1 Gag and examined the packaging efficiencies of RNA derived from these two HIV-2 proviruses. WT-17T and PIH have similar general structures, containing all cis-acting elements required for viral replication and expressing Tat and Rev (Figure 1C). However, both constructs contain inactivating mutations in the gag gene to abolish Gag/Gag-Pol expression. Additionally, these two constructs express marker genes in nef: WT-17T expresses the mouse thy1.2 gene, whereas PIH expresses a puromycin resistance gene and a mouse heat stable antigen (hsa) gene. The translation of the hsa gene is facilitated by the internal ribosomal entry site (IRES) from the encephalomyocarditis virus (Figure 1C). The WT-17T also contains two additional features. First, there is a 12- nt insertion near the 3′ end of the gag gene that generates a polymorphic site with the PIH construct and allows for the use of allele-specific PCR to detect and distinguish between RNAs from these two viruses. Secondly, WT-17T has a modified dimerization initiation signal, changing GGTACC to GCGCGC; this alteration reduces the likelihood of WT-17T RNA dimerizing with PIH RNA.

**Figure 2:**
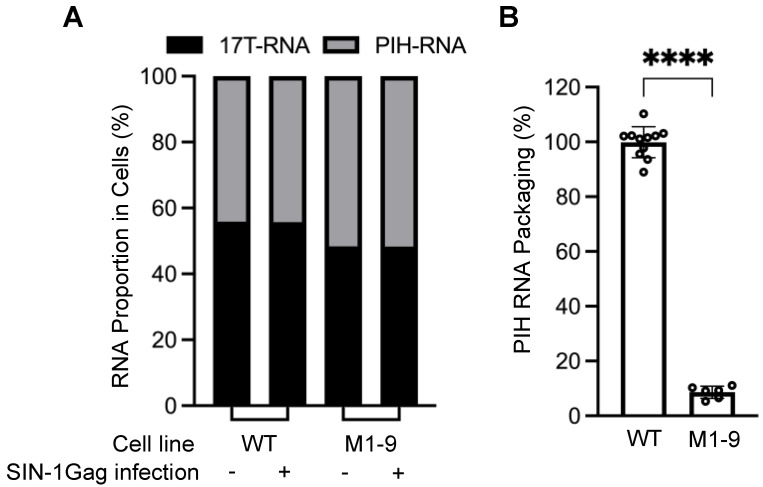
Effect of mutating unpaired guanosines in the HIV-2 5′ UTR on RNA expression and packaging by HIV-1 Gag. (**A**) RNA proportion of two HIV-2 proviruses in the cytoplasm. WT, both proviruses contain wild-type 5′ UTR. M1-9 contains guanosine substitutions in all 9 sites within the 5′UTR. 17T-RNA, RNA from WT-17T. (**B**) The role of unpaired guanosines on RNA packaging by HIV-1 Gag. Results shown are the averages of at least three independent experiments. Data are depicted as the mean ± standard deviation (S.D.); open circles indicate the values obtained from each experiment, each with at least one data point. PIH RNA packaging efficiency calculated as described in Figure 1D. *p*-values were calculated by an unpaired t-test with Welch’s correction; ****, *p* < 0.0001.

Stable cell lines expressing the two HIV-2 proviruses were generated by sequential infection (Figure 1D). These cell lines consist of cells with predominantly one WT-17T provirus and one PIH provirus. To introduce HIV-1 Gag, we infected the cell lines with a self-inactivating lentivirus, SIN-1Gag, that expresses HIV-1 Gag from an internal cytomegalovirus (CMV) promoter (Figure 1C,D). The 3′ LTR of SIN-1Gag contains a deletion that removes most of the U3 (ΔU3; Figure 1C). During reverse transcription, the U3 from the 3′ end of the genome is used as a template to generate the LTRs, resulting in both LTRs containing the ΔU3 deletion, thereby inactivating transcription from the lentiviral LTR promoter. Hence, after infecting cells, the SIN-1Gag provirus expresses HIV-1 Gag from the internal CMV promoter but does not express HIV-1 RNA with a packaging signal and should not interfere with the packaging of the HIV-2 RNAs in this system. To validate our experimental system, we used a cell line that expressed two proviruses, WT-17T and PIH-WT, both with all the guanosines in the 5′ UTR. We isolated RNAs from cells and performed quantitative allele-specific RT-PCR to determine the amounts of RNAs from WT-17T and from PIH-WT. This method only measures the unspliced HIV-2 RNA because it detects the polymorphic site located at the 3′ end of the gag gene. We found that these two proviruses were expressed at similar levels in cells; PIH-WT RNA comprised ~44% of the total HIV-2 unspliced RNA (WT-17T plus PIH-WT) (Figure 2A; WT, without SIN-1Gag). The ratios of WT-17T and PIH-WT RNA expression did not change when HIV-1 Gag was introduced into the cell line via lentiviral transduction with SIN-1Gag (Figure 2A; WT with and without SIN-1Gag). We also harvested viruses produced from SIN-1Gag-transduced cells, measured the amounts of the two HIV-2 RNAs, and calculated the packaging efficiency of PIH RNA. The efficiency is defined as the proportion of PIH RNA in the HIV-2 virion divided by the proportion of PIH RNA in cells [(PIH RNA in virions/total HIV-2 RNA in virions)]/[(PIH RNA in cells/total HIV-2 RNA in cells)]. If the proportion of PIH RNA in virions reflects that in cells, then the packaging efficiency would be close to 100%. Our results showed that RNA from WT-PIH is packaged close to 100% (100 ± 6%; Figure 2B; WT).

### 3.2. Unpaired Guanosines in the HIV-2 5′ UTR Are Required for HIV-1 Gag-Mediated RNA Packaging

We generated the PIH-based M1-9 mutant, in which 18 guanosines in 9 sites were mutated by substitution (Figure 1A,B), and examined the expression and packaging efficiency of the M1-9 mutant by HIV-1 Gag (Figure 1D). Our results showed that the mutant provirus was expressed at a level similar to that of the WT provirus in cells; M1-9 RNA constituted 52% of the total unspliced HIV-2 RNA in cells (Figure 2A). However, M1-9 RNA was packaged poorly, with an efficiency of 9 ± 2% (Figure 2B). Thus, similar to HIV-2 Gag, HIV-1 Gag also requires unpaired guanosines in the HIV-2 5′ UTR for efficient RNA packaging.

**Figure 3:**
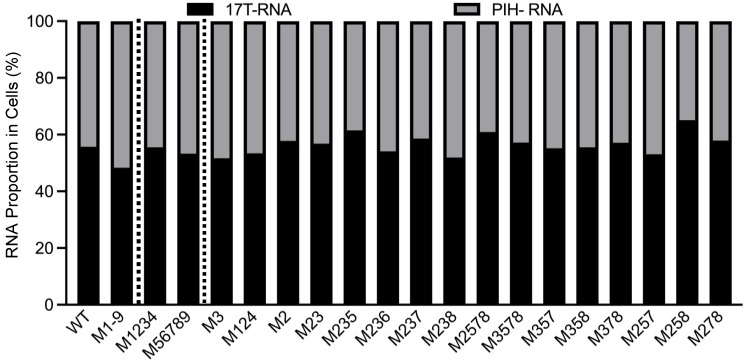
Proportions of cytoplasmic RNA expressed by WT-17T (17T-RNA) and PIH proviruses from WT and mutants cell lines. *X*-axis labels reflect mutations in the PIH proviruses. WT and M1-9 results are from Figure 2A, shown here for comparison. Results shown are the averages of at least three independent experiments.

**Figure 4:**
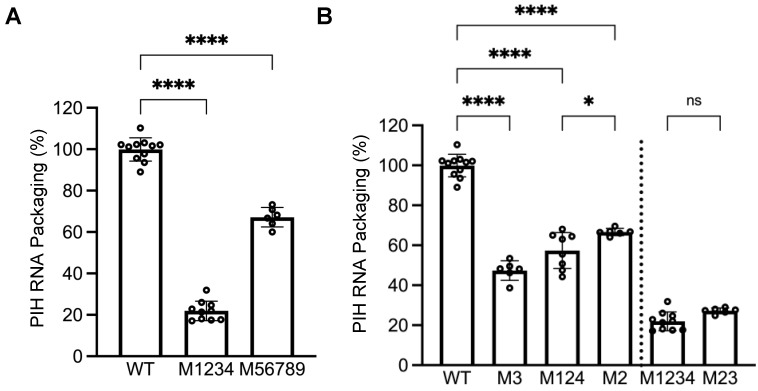
Identification of unpaired guanosines in the HIV-2 5′ UTR important for packaging by HIV-1 Gag. (**A**) Effects of guanosine substitutions in the first four (M1234) or last five (M56789) sites on RNA packaging. (**B**) Elements in sites 1 to 4 that are important for RNA packaging. Results shown are the mean ± S.D. of at least three independent experiments. Open circles indicate values obtained from each experiment. WT results from Figure 2B are shown here as a comparison. *p*-values were calculated by a one-way ANOVA with Bonferroni correction for multiple pairwise comparisons. *, *p* < 0.05; ****, *p* < 0.0001; ns, not significant.

To delineate mutations in M1-9 that cause packaging defects, we generated two PIH-based mutants, one in which the first four sites (M1234) were mutated and the other in which the last five sites (M56789) were mutated. Our allele-specific RT-PCR analyses showed that the expression levels of these two mutant proviruses were similar to that of WT proviruses (Figure 3). However, their RNA genome packaging efficiencies were compromised; M1234 RNA was packaged at 22 ± 5%, whereas M56789 RNA was packaged at 67 ± 5% (mean ± SD) (Figure 4A). Thus, both sets of mutations decreased RNA packaging by HIV-1 Gag, indicating that each set contains unpaired guanosines important for HIV-1 Gag/HIV-2 RNA interactions.

### 3.3. Unpaired Guanosines in Sites 2 and 3 Play Major Roles in HIV-1 Gag-Mediated HIV-2 RNA Packaging

To further delineate sites within mutant M1234 that are necessary for HIV-1 Gag–HIV-2 RNA interactions, we generated PIH-based mutants M3 and M124, in which site 3 or sites 1, 2, and 4 were mutated, respectively. Our analyses demonstrated that both mutant proviruses expressed unspliced RNA at levels similar to WT viruses (Figure 3). However, these mutant RNAs exhibited packaging defects; packaging efficiencies for M3 RNA and M124 RNA were 47 ± 5% and 57 ± 9%, respectively (Figure 4B). Site 3 was previously identified as the sole primary site for HIV-2 Gag-mediated RNA packaging [26]. Thus, our results indicate that the unpaired guanosines in site 3 play an important role in interacting with both HIV-1 Gag and HIV-2 Gag. In contrast, our previous study showed that mutating sites 1, 2, and 4 did not affect the packaging efficiency of HIV-2 Gag, which is distinct from our results here using HIV-1 Gag (Figure 4B). To better delineate the site(s) important for HIV-1 Gag recognition, we generated PIH-based M2, in which the unpaired guanosines in site 2 were replaced with adenosines. Our results showed that M2 provirus RNA was expressed in cells at a level similar to RNA with WT 5′ UTR (Figure 3) but packaged at a significantly reduced level by HIV-1 Gag (67 ± 2%; Figure 4B). These results indicate that both site 2 and site 3 are required for efficient packaging by HIV-1 Gag, which is different from HIV-2 Gag. We then generated PIH-based M23, in which the guanosines in sites 2 and 3 are mutated. Our results showed that RNA from M23 was packaged at 27 ± 1% by HIV-1 Gag (Figure 4B), which is not significantly different from that of M1234, indicating that most of the packaging defects of M1234 come from mutation of sites 2 and 3.

### 3.4. Unpaired Guanosines in Sites 5, 7, and 8 Also Play a Role in HIV-1 Gag-Mediated HIV-2 RNA Packaging

**Figure 5:**
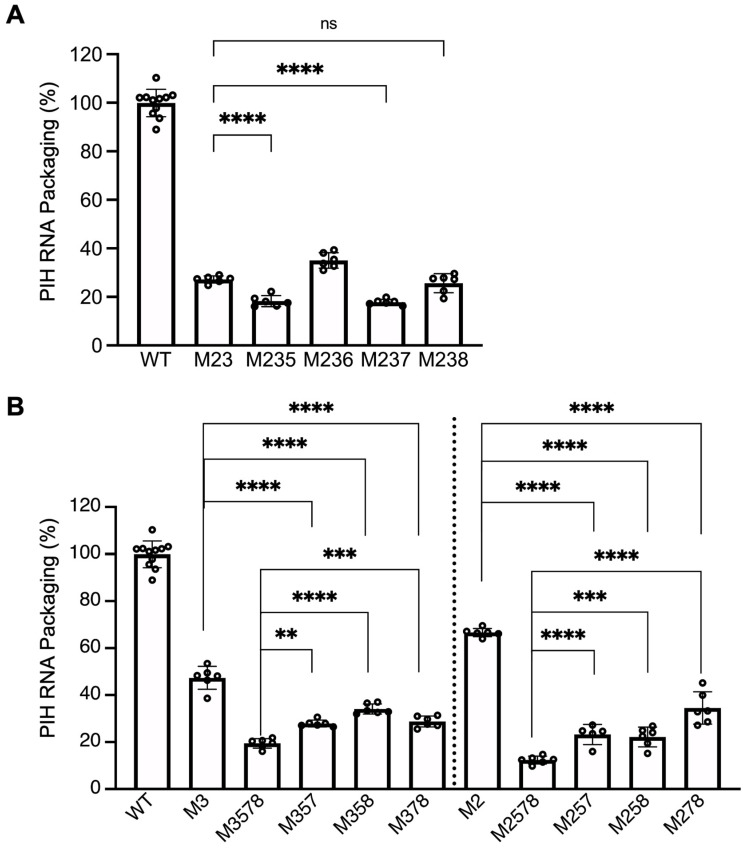
Delineating the role of guanosines in sites 5, 6, 7, and 8 on RNA packaging. (**A**) Effects of adding substitutions from sites 5, 6, 7, or 8 to mutants with site 2 and 3 mutations. (**B**) Effects of mutating site 3 or site 2 in combination with the indicated sites on RNA packaging. WT and M1-9 results from Figure 2B and M2, M3, and M23 results from Figure 4B are shown here as a comparison. Results shown are the mean ± S.D. of at least three independent experiments. Open circles indicate values obtained from each experiment. *p*-values were calculated by a one-way ANOVA with Bonferroni correction for multiple pairwise comparisons. **, *p* < 0.0021; ***, *p* < 0.0002; ****, *p* < 0.0001; ns, not significant.

As shown in Figure 4A, mutating the last five sites (5, 6, 7, 8, 9) caused a significant decrease in RNA packaging compared to WT. To define the role of these guanosines in HIV-2 RNA packaging mediated by HIV-1 Gag, we added an additional mutated site to M23 to generate M235, M236, M237, and M238 and determined whether these added mutations would further decrease RNA packaging compared to the M23 mutant. Site 9 was not included as it did not impact packaging mediated by HIV-2 Gag [26]. Our result showed that M235 and M237 were packaged at 18 ± 2% and 18 ± 1%, respectively, efficiencies that were lower than M23 (Figure 5A). In addition, M238 is packaged at 26 ± 4%, which is slightly lower (although not statistically significant) than M23. In contrast, M236 RNA was packaged at 35 ± 3%, which is not lower than that of M23. These results indicate that sites 5, 7, and possibly site 8 may interact with HIV-1 Gag.

### 3.5. Site 2 Exerts More Impact in HIV-1 Gag-Mediated than HIV-2 Gag-Mediated Packaging of HIV-2 RNA

**Figure 6:**
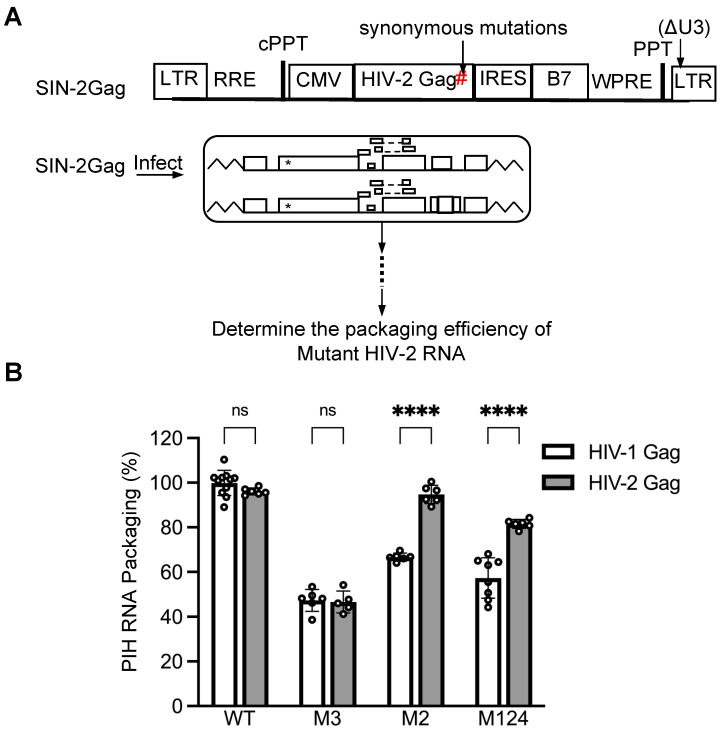
Comparison of the same RNA substrates packaged by HIV-1 or HIV-2 Gag. (**A**) General structures of the lentiviral vector expressing HIV-2 Gag and experimental protocol. *, indicate inactivating mutations in *gag*; #, synonymous mutations in the HIV-2 gag gene to avoid detection by the allele-specific RT-PCR. (**B**) Comparisons of the packaging efficiencies of HIV-1 or HIV-2 Gag. HIV-1 Gag packaging results (open bars) from Figure 2 (WT) and 4 (M2, M3, and M124) are shown as a comparison. Results are the mean ± S.D. of at least three independent experiments. Open circles indicate values obtained from each experiment. *p*-values were calculated by a two-way ANOVA with Bonferroni correction for multiple comparisons. ****, *p* < 0.0001; ns, not significant.

In the current study, we identified two primary sites, sites 2 and 3, in the HIV-2 5′ UTR that are essential for efficient HIV-1 Gag-mediated packaging. In contrast, our previous study demonstrated that site 2 is not necessary for efficient HIV-2 Gag-mediated encapsidation [26]. These two studies used different methods to measure packaging efficiencies; the previous study used HIV-2 constructs that produce both Gag and unspliced RNA, and RNA packaging was quantified by imaging RNA incorporated in individual viral particles [26]. In the current study, stable cell lines were generated, and mutant RNA packaging was measured in the presence of a competing RNA containing a wild-type 5′ UTR. To ensure that the difference we observed was not due to the distinct experimental systems, we expressed HIV-2 Gag in the current experimental system and examined the HIV-2 Gag-mediated packaging of HIV-2 RNA (Figure 6A). Briefly, we replaced the HIV-1 gag gene in the lentiviral vector with the HIV-2 gag gene to generate SIN-2Gag. Additionally, we introduced synonymous mutations in the HIV-2 gag gene (shown as # in Figure 6A) to avoid the detection of SIN-2Gag RNA by the allele-specific RT-PCR assay.

We first examined the cell line expressing WT-17T and PIH RNA containing the WT 5′ UTR. As expected, both HIV-1 and HIV-2 Gag polyproteins packaged PIH RNA efficiently (Figure 6B). The M3 mutant also yielded similar results for both HIV-1 and HIV-2 Gag-mediated RNA packaging, consistent with our observations that site 3 is a primary site for both Gag polyproteins (Figure 6B). In contrast, the M2 mutant was packaged efficiently by HIV-2 Gag (95 ± 4%) but exhibited a defect when encapsidated by HIV-1 Gag (67 ± 2%). Similarly, M124 exhibited less defects when packaged by HIV-2 Gag than by HIV-1 Gag (Figure 6B). These results confirmed that site 2 is more important in mediating HIV-1 Gag packaging than HIV-2 Gag packaging.

## 4. Discussion

HIV-1 and HIV-2 both package their own RNA genomes efficiently. However, the mechanisms by which Gag specifically selects and packages unspliced viral RNAs from an abundant pool of cellular mRNAs are not fully understood. In this report, we demonstrated that multiple guanosines in the HIV-2 5′ UTR are required for HIV-1 Gag-mediated RNA packaging. Furthermore, HIV-1 Gag can recognize the same set of guanosines used by HIV-2 Gag, thereby allowing the cross-packaging of HIV-2 RNA. Nevertheless, there are two main differences between the HIV-1 and HIV-2 Gag protein interactions with HIV-2 RNA. First, HIV-1 Gag has two primary binding sites whereas HIV-2 has one. A primary binding site is defined as the substitution of guanosines within this site alone resulting in packaging defects. Mutating guanosines in site 2 causes defects in HIV-1 Gag-mediated but not HIV-2 Gag-mediated RNA packaging. Interestingly, there are two primary binding sites in the 5′ UTR of HIV-1 RNA [25], suggesting the possibility that the initial binding stoichiometry of HIV-1 Gag and HIV-2 Gag on RNA substrates may differ. Additionally, cumulative mutations in HIV-2 5′ UTR have different effects on HIV-1 Gag- and HIV-2 Gag-mediated packaging. We have previously shown that combining guanosine substitutions caused synergistic defects in the HIV-2 Gag-mediated packaging of HIV-2 RNA. In contrast, combining two primary site mutations has additive effects on HIV-1 Gag-mediated HIV-2 RNA packaging. For example, the packaging efficiencies of M2 and M3 are 67% and 47%, respectively; their additive effect (47% × 67% = 31%) is similar to the packaging efficiency of M23 (27%; Figure 4B). Similarly, the packaging efficiencies of M3 and M124 are 47% and 57%, respectively, and the expected additive effect (47% × 57% = 27%) is similar to the packaging efficiency of M1234 (22%) (Figure 4B). Thus, combining these guanosine substitutions in HIV-2 5′ UTR appears to have additive and not synergistic effects on HIV-1 Gag mediated packaging. Interestingly, in the context of packaging HIV-1 RNA, HIV-1 Gag also displays synergistic defects when combining guanosine mutations in the HIV-1 5′ UTR [25]. The cis- and trans-acting elements from the same virus coevolved to optimize Gag/RNA interactions. It is likely that the interactions of a pair of heterologous elements, such as HIV-1 Gag and HIV-2 RNA, are not optimized, leading to the lack of synergistic effects.

The preferential binding sites of Gag proteins on the viral RNA genome have been mapped in several viruses [27,28,35,36,37,38,39]. The NC protein of murine leukemia virus (MLV) preferentially binds a single-stranded UCUG-UR-UCUG RNA sequence with the strongest binding on the first U and G of the UCUG motif. Mutating the guanosines in these motifs caused a drastic decrease in MLV RNA packaging [35]. A UCUG sequence in the 5′ UTR of the feline immunodeficiency virus has been identified as a putative Gag binding site; mutating this site reduced Gag binding in vitro [37]. In these two viruses, the high-affinity binding sites for Gag are within the UCUG sequence. In contrast, preferential HIV-1 Gag binding sites are not within a conserved sequence, such as UCUG, but are exposed, single-stranded guanosines in the RNA secondary structure [27,28]. Substituting these guanosines caused defects in RNA genome packaging [25,28]. Here, we showed that the unpaired guanosines in HIV-2 5′ UTR are also important for the HIV-1 Gag-mediated packaging. Substituting guanosines in either site 2 or site 3 causes significant packaging defects (Figure 4B). These guanosines are not within conserved primary sequences: their sequences are AGUAAG (site 2) and GGAGU (site 3). Guanosines in several sites, when combined, can cause defects in HIV-1 Gag-mediated packaging. The guanosines in site 5 (AGAAGAG), site 7 (AAAAAUGU..//..UGCUAUC), and site 8 (GAAAG) are also not within a discrete primary sequence. These results demonstrate that the preferential binding sites for HIV-1 Gag do not need to be in a conserved primary sequence but are single-stranded guanosines in the RNA structures. It is noteworthy that the Gag or NC binding sites of the human T cell leukemia virus type 1 (HTLV-1) [39], mouse mammary tumor virus (MMTV) [36], and Mason–Pfizer monkey virus (M-PMV) [37] have also been identified. Using cross-linking combined with chemical probing, the HTLV-1 NC binding sites have been mapped to a GAGAG and a GAGC sequence, both in a stem-loop region [39]. Using binding and footprinting assays, MMTV and M-PMV Gag proteins bind a purine loop in the 5′ UTR of their unspliced RNA, although the sequence of the loop is different [36,37]. While Gag/NC from multiple retroviruses appears to bind guanosines/purine-rich sequences in single-stranded regions, it is very likely that there are differences among these sites in the RNAs to facilitate packaging by their own Gag proteins.

In this report, we demonstrated that HIV-1 and HIV-2 Gag can recognize the same sets of guanosines in the HIV-2 5′ UTR for genome packaging. These findings suggest that Gag needs to recognize the binding sites in the heterologous viral RNA for cross-packaging to occur. However, HIV-2 Gag does not efficiently package HIV-1 RNA, and the nonreciprocal nature of this cross-packaging remains incompletely understood. An HIV-1-based chimeric virus with the HIV-1 NC domain replaced by the counterpart from HIV-2 has RNA packaging defects [40]. However, a 2-amino acid substitution in the HIV-2 NC domain restored most of the RNA packaging for the hybrid virus [40]. These findings suggest that there may be suboptimal HIV-2 NC/HIV-1 RNA interactions. We have previously proposed that viral RNA packaging is the nucleation event of virus assembly: HIV-1 RNA provides preferred Gag binding sites and facilitates Gag/Gag interactions to promote virus assembly [41]. The inability of HIV-2 Gag to package HIV-1 RNA could come from two Gag/RNA interaction defects. First, it is possible that there are subtle context differences between the exposed guanosines in the two 5′ UTRs, causing HIV-2 Gag to be unable to sufficiently interact with sites in HIV-1 RNA. It is also possible that HIV-1 RNA does not provide the correct platform to promote HIV-2 Gag multimerization to nucleate virus assembly. These two possibilities are not mutually exclusive. Future studies are needed to determine the mechanisms by which HIV-2 Gag is unable to package HIV-1 RNA.

## Data Availability

All the data are contained within the article.
